# Testing of Reverse Causality Using Semi-Supervised Machine Learning

**DOI:** 10.1017/psy.2025.13

**Published:** 2025-04-07

**Authors:** Nan Zhang, Heng Xu, Manuel J. Vaulont, Zhen Zhang

**Affiliations:** 1 Department of Management, Warrington College of Business, University of Florida, Gainesville, FL, USA; 2 Management and Organizational Development Group, D’Amore-McKim School of Business, Northeastern University, Boston, MA, USA; 3 Department of Management, Strategy and Entrepreneurship, Edwin L. Cox School of Business, Southern Methodist University, Dallas, TX, USA

**Keywords:** machine learning, reverse causality, semi-supervised learning

## Abstract

Two potential obstacles stand between the observation of a statistical correlation and the design (and deployment) of an effective intervention, *omitted variable bias* and *reverse causality*. Whereas the former has received ample attention, comparably scant focus has been devoted to the latter in the methodological literature. Many existing methods for reverse causality testing commence by postulating a structural model that may suffer from widely recognized issues such as the difficulty of properly setting temporal lags, which are critical to model validity. In this article, we draw upon advances in machine learning, specifically the recently established link between causal direction and the effectiveness of semi-supervised learning algorithms, to develop a novel method for reverse causality testing that circumvents many of the assumptions required by traditional methods. Mathematical analysis and simulation studies were carried out to demonstrate the effectiveness of our method. We also performed tests over a real-world dataset to show how our method may be used to identify causal relationships in practice.

## 1 Introduction

A fundamental purpose of research in psychology—and many other disciplines in social sciences for that matter—is to understand causal relationships between variables. In particular, it is both theoretically and practically important to distinguish between the mere observation of associations (between variables) and cases where causality can be inferred. When controlled randomized experiments are impractical, how to properly do so has garnered considerable attention in multiple disciplines (e.g., Pearl, 1998) including psychology (Rogosa, 1980). The practical significance of this inquiry becomes apparent considering the pivotal role that causal inference plays in formulating effective intervention strategies. For example, research has consistently shown that individuals who occupy central positions in their social networks (i.e., *centrality*) tend to receive more favorable assessment of *charisma* from their connections (Balkundi et al., 2011). Yet, the mere identification of this correlation^1^ does not permit the conclusion that enhancing an individual’s social-network centrality would be an effective intervention for boosting their charisma. As a case in point, whereas some scholars view charisma as an outcome of social-network patterns (Pastor et al., 2002), others contend that charisma is what attracts followers and allows an individual to occupy a central position in the first place (Shils, 1965), suggesting that the aforementioned intervention might be less effective.

Two potential obstacles stand between (a) the observation of a robust correlation between two constructs *X* and *Y*, like the centrality-charisma correlation, and (b) the effectiveness of deploying an intervention on *X* to change *Y*, like an attempt to boost centrality in order to increase charisma (Bollen, 1989, p. 41). The first is *omitted variable bias* (Mauro, 1990), meaning that an unobserved confounder induces the co-variation of both *X* and *Y*. For example, individuals high on extraversion may excel on both social-network centrality and charisma. The other is *reverse causality* (Leszczensky & Wolbring, 2022), meaning that the causal influence flows in the reverse direction (i.e., 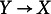 ) from what is required by the intended intervention—e.g., charisma influences centrality but not the other way around. There is a substantial body of methodological research on assessing the magnitude of omitted variable bias in a relationship (e.g., Busenbark et al., 2022; Cinelli & Hazlett, 2020; Harring et al., 2017; Mauro, 1990). Yet comparatively little attention has been directed toward testing for reverse causality (Leszczensky & Wolbring, 2022). Admittedly, the issue may not be applicable in situations where a clear causal direction is obvious (e.g., cancer 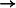 age is implausible). Nonetheless, for psychological constructs, concerns on reverse causality are prevalent, especially when theoretical arguments could be made for both directions. The focus of this work is to develop a method for testing reverse causality in observational data, with an emphasis on panel data.

To empirically investigate the issue of reverse causality in observational data, some existing methods, like cross-lagged panel models (CLPM; Hamaker et al., 2015), commence by presuming a reciprocal relationship, before postulating a structural model based on the assumption and empirically estimating the model to assess the magnitude in both directions while considering auto-regressive effects. Some other methods infer the direction of causality by exploiting certain distributional and/or functional features assumed of the underlying data-generating process, such as non-normality in a linear model (e.g., Shimizu et al., 2006; von Eye & DeShon, 2012), statistical independence between input variables and additive noise (e.g., Hoyer et al., 2008; Rosenström et al., 2023), etc. These methods pose two unsolved issues. First, from a theoretical perspective, given that many psychological theories (and theories in other fields) stipulate unidirectional (instead of reciprocal) relationships, there exists substantial theoretical interest in *testing* the direction of causality rather than assuming it away with the adoption of a reciprocal model like CLPM. For example, the structural advantage theory of social networks (Brass, 1984; Burt, 1992) posits a unidirectional effect of one’s social network characteristics (e.g., centrality) on individual behaviors (e.g., charismatic leadership). Given the robust correlation reported in the literature for centrality and charisma (e.g., Balkundi et al., 2011), a well-powered empirical study that shows the absence of a reverse effect (i.e., charisma 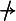 centrality) would provide strong evidential value for supporting the structural advantage theory. Second, from a methodological perspective, formulating an appropriate model—or accurately specifying its distributional/functional features (e.g., whether it is linear)—can be particularly challenging when dealing with panel data (Hamaker, 2024; Lucas, 2023). For example, the validity of many existing panel models (or even the notion of Granger causality itself) is known to break down when the sampling frequency is improperly specified (Shojaie & Fox, 2022; Vaisey & Miles, 2017), when there are confounders that are unaccounted for (e.g., Hamaker et al., 2015), etc.

A promising avenue for addressing these issues of existing methods arises from causal learning (Peters et al., 2017), a branch of machine learning that injects causal inference into the design of learning algorithms. Central to this approach is a fundamental question: if we were to train a machine learning model that predicts *Y* from *X* (based on a limited number of training data points 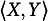 ), could knowledge about the probability distribution of *X* (i.e., 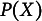 ) help us improve the predictive accuracy of the trained model? Schölkopf et al. (2012) show that the answer is positive *if and only if* the causal direction flows from *Y* to *X*. This suggests that, by demonstrating a machine learning model’s ability to capitalize on 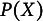 for enhancing predictive accuracy toward *Y*, we would identify the existence of reverse causality. Even more importantly, this proposition would hold irrespective of the functional form of the *X*–*Y* relationship.

Whereas Schölkopf et al. (2012) establish the dependence between causal direction and the predictive accuracy of semi-supervised learning algorithms, they leverage this finding to understand why semi-supervised learning works over some datasets but not others, instead of developing a concrete method for testing reverse causality. In the current research, we address this gap by developing a novel method for testing reverse causality over panel data. The contribution of our work is two-fold. First, our method for reverse causality testing allows researchers to rigorously test their causal theories by addressing two issues that researchers face. On the one hand, our method allows researchers to directly test unidirectional relationships, instead of assuming these relationships are reciprocal in nature. On the other hand, our method does not require the specification of distributional or functional features such as the sampling frequency or the shape of the proposed relationship. By demonstrating the effectiveness of our method using simulation studies and a case study, we show its applicability for psychology researchers in theory building and testing. Second, our work pioneers the integration of advancements in causal learning into the methodological arsenal of psychology for causal inference. We present conceptual arguments and mathematical formalization that link reverse causality testing with the predictive accuracy of semi-supervised learning (Van Engelen & Hoos, 2020). In doing so, we enrich the understanding of how machine learning could contribute to the psychological methods literature (e.g., Sterner et al., 2023; Wilcox et al., 2023; Zimmer & Debelak, 2023) and open up future avenues of inquiry at the intersection of psychological research and computer science.

## 2 Literature review

In this section, we briefly review the existing literature on reverse causality testing and the machine learning method we propose to use (i.e., semi-supervised learning).

### 2.1 Reverse causality testing

Compared with the rich and growing literature on omitted variable bias—in psychology (Harring et al., 2017), sociology (Halaby, 2004) and economics (Wüthrich & Zhu, 2023)—researchers across disciplines have made relatively limited progress on the testing of reverse causality over panel data. As summarized by Leszczensky & Wolbring (2022), a common approach is to specify causal direction in a panel model by applying *temporal lags* on variables that represent the “cause” after partialing out auto-regressive effects. For example, a causal direction of 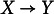 would be reflected by setting a lagged (e.g., previous-wave) value of *X* and the contemporary value of *Y* as independent and dependent variables, respectively, in the panel model, suggesting that *X* has a causal, lagged, effect on *Y*. A variety of panel models follow this idea (Orth et al., 2021), such as lagged first-difference (LFD) models (Vaisey & Miles, 2017), CLPM (Hamaker et al., 2015), etc.

As these existing panel models rely on temporal lags to identify the causal direction, how to specify the amount of this temporal lag becomes a prominent question. Theoretically deriving the “correct” temporal lag is obviously even more challenging than discerning the causal direction, suggesting the need for methods to be robust to misspecified temporal lags. Unfortunately, Vaisey & Miles (2017) show that panel models such as LFDs can be highly sensitive to the misspecification of temporal lags. Leszczensky & Wolbring (2022, Figure 2) also demonstrate that, when a contemporaneous effect is mis-characterized as lagged in a CLPM, the model can produce highly biased estimates. More fundamentally, the very notion of Granger causality, which underpins all panel models, is known to break down with a mis-specified temporal lag (Shojaie & Fox, 2022).

Beyond panel models, other existing methods for reverse causality testing rely on certain distributional/functional features assumed of the underlying data-generating process. Some—like Direction Dependence Analysis (DDA; Li & Wiedermann, 2020; Pornprasertmanit & Little, 2012; von Eye & DeShon, 2012; Wiedermann & Li, 2018) and Linear Non-Gaussian Acyclic Models (LiNGAM; Shimizu et al., 2006)—exploit the non-normality of data distributions in a linear model to infer causal direction. In the case of DDA, for example, the causal direction may be inferred by comparing the degrees of departure^2^ from normality across different variables. For longitudinal data, extensions of these methods (e.g., Bauer et al., 2016; Geiger et al., 2015; Hyvärinen et al., 2010) leverage the non-normality of noise to identify causal direction in multivariate time series. Additionally, methods like Rosenström et al.’s (2023) directional analysis approach, additive noise models (Hoyer et al., 2008; Peters et al., 2014) and (more generally) post-nonlinear models (Zhang & Hyvärinen, 2009) infer causal direction by assuming statistical independence between the input variables and an additive noise component within linear or nonlinear models. Yet, like panel models’ reliance on properly specified temporal lags, these methods also hinge on accurately defining certain distributional or functional features of the relationship between variables—knowledge that may not be available *a priori*.

These issues of existing methods raise an important question: Can we test for reverse causality *without* constraining the functional form of the data-generating process? Doing so would not only circumvent the complexities of specifying the temporal lag, but also relax the linearity or additive noise assumptions that permeate existing methods. We seek to answer this question in the current work by integrating recent advances in machine learning and the broader causal inference literature.

### 2.2 Semi-supervised learning

The objective of semi-supervised learning is to approximate the function *f* that links independent variables *X* to a dependent variable *Y* such that 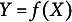 . This is done by learning from two datasets containing i.i.d. samples. The first dataset, typically known as *labeled set*, provides 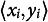 (i.e., paired *X*–*Y* values) for 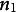 data points. The second dataset provides only the values of *X*, but *not Y*, for other 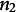 data points, and is therefore known as the *unlabeled set*. In machine learning, it is generally assumed that 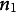 is much smaller than 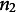 , due mainly to the high cost of acquiring *Y* in practice. For example, a task for which semi-supervised learning has shown remarkable success is image classification (Xie et al., 2020). With this task, *X* is an image and *Y* is its category (e.g., landscape, portrait). It is virtually cost-less to collect millions of images from the web, but considerably more expensive to hire human workers to properly label the collected images. In this case, we may opt to manually label only a few observations of *X*, leading to the assumption that 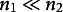 .

The limited size of the labeled set means that, if we were to launch a canonical supervised learning algorithm (e.g., OLS or logistic regression), which can only learn from the labeled set, we would not be able to obtain an accurate prediction of *Y*. The uniqueness of semi-supervised learning lies in its ability to leverage the 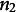 unlabeled, *X*-only, data points to significantly improve the predictive accuracy of the learned 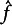 . To this end, numerous methods have been proposed for semi-supervised learning (Van Engelen & Hoos, 2020). A famous example, which we will further elaborate later in the article, is self-training (e.g., Sohn et al., 2020), which can be readily integrated with many supervised learning algorithms such as logistic regression. With self-training, we start by running a supervised learning algorithm (e.g., logistic regression) over the 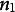 labeled data points to generate an approximation of *f*, denoted by 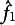 , which predicts *Y* based on an input *X*. Then, we apply 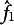 over each of the 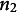 unlabeled data points to predict its *pseudo-label* (i.e., an estimate of *Y*). Note that when a machine learning model is used for prediction, it may generate not only a point-estimate (e.g., binary prediction in logistic regression) but also a confidence level associated with the estimate (e.g., log-odds in logistic regression). Leveraging this, we select from the 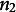 pseudo-labels those with confidence above a pre-determined threshold, add their *X* values paired with pseudo-labels (i.e.,) 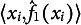 to the labeled set, before running the supervised learning algorithm again to update our approximation of *f*. This process can continue iteratively until no more pseudo-labels can be added.

Whereas the efficacy of semi-supervised learning has long been established (Van Engelen & Hoos, 2020), there has been limited research probing *why* unlabeled data, which lack any information about *Y*, can bolster our understanding of the *X*–*Y* relationship. To this end, Buja et al. (2019) establish that the distribution of a variable *X* may convey rich information about the *X*–*Y* relationship when the relationship is not strictly linear. For the high-dimensional case where *X* comprises multiple variables, Niyogi (2013) attributes the success of semi-supervised learning to the concept of manifold regularization (Belkin et al., 2006). At its core, Niyogi (2013) posits that almost all machine learning algorithms predicate their predictions on a smoothness assumption: if two data points are similar in *X*, then they also tend to be analogous in *Y*. Unfortunately, defining “similar” in the context of multi-dimensional *X* is challenging—and requires structural insights into the multivariate distribution of *X*—because common similarity measures like Euclidean distance are known to become meaningless in high-dimensional spaces (Aggarwal et al., 2001). By providing a more refined depiction of the multi-dimensional distribution of *X*, unlabeled data allow us to more accurately estimate the similarity of two data points in *X*, thereby enhancing our predictive precision for *Y* (Niyogi, 2013).

While this line of research sheds light on the mechanics of semi-supervised learning, it does not address why semi-supervised learning achieves high predictive accuracy with certain datasets but performs poorly with others (Schölkopf et al., 2012). The missing piece—the key to understanding the conditions under which semi-supervised learning succeeds—lies in the causal effects driving the generation of the observed data, as we will elaborate in the next section.

## 3 Linking causality with semi-supervised learning

In this section, we draw upon insights from causal learning (Peters et al., 2017)—specifically Janzing & Schölkopf’s (2015) mathematical characterisation of Schölkopf et al.’s (2012) seminal finding on the working condition for semi-supervised learning—to elucidate how the predictive accuracy of semi-supervised learning over panel data may reveal the existence of reverse causality in the underlying data-generating process. Our explanation unfolds in three steps. First, we offer conceptual arguments through an illustrative example. Following this, we delve into mathematical formalization, analyzing a special case where the data-generating process features linear, cross-lagged, effects. Finally, we expand our discussions to justify the reasoning behind a test that can be applied to any arbitrary data-generating process. The specific computational algorithms for semi-supervised learning will be described in the next section.

### 3.1 Conceptual illustration

In what follows, we offer an intuitive explanation for the key insight of Schölkopf et al. (2012) through two observations: (1) 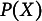 is useless for predicting *Y* if 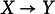 , and (2) if 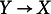 , 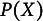 (when combined with a small labeled set) may lead to an accurate prediction model toward *Y*. The first is obvious: When 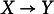 fully characterizes the *X*–*Y* relationship, we can represent their relationship as a stochastic function *f* such as 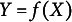 . Changing *f* clearly has no impact on 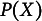 . Consequently, knowledge of 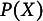 reveals no information about *f*, making it useless for the prediction of *Y* (Janzing & Schölkopf, 2010).

**Figure 1:**
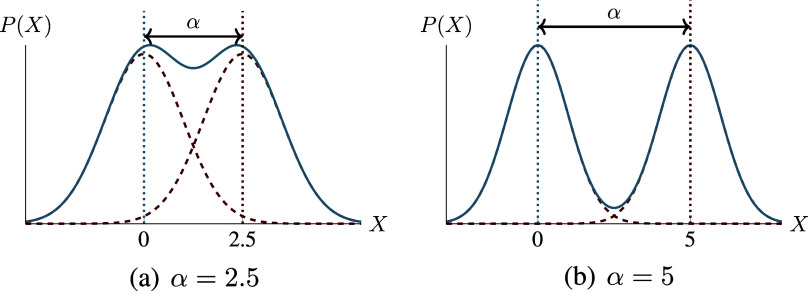
Illustrative example for 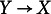 .*Note*: Both panels depict the probability density function of 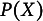 when 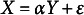 , where *Y* follows Bernoulli distribution with 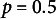 and 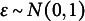 . Note that, in either case, 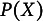 follows a Gaussian mixture distribution with two equal-weight components, which are illustrated in red dashed lines. The mean difference between the two Gaussian components is always equal to 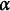 , suggesting that the functional relationship between *X* and *Y* can be precisely inferred from 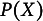 .

For the second observation, consider a simple example of 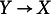 depicted in Figure 1, where 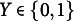 is binary and 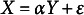 with 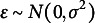 . In this case, altering the causal mechanism (i.e., 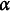 ) obviously modifies 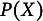 . From the perspective of machine learning, this means that 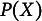 now encapsulates certain cues regarding 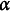 , offering the potential for building an accurate prediction model toward *Y*. As can be seen from Figure 1, for this specific example, knowledge of 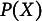 alone permits an exact inference^3^ of 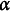 , which is all that is required to build a Bayes-optimal predictior of *Y*.

More generally, Schölkopf et al. (2012) contend that 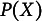 is “independent” of *f* if reverse causality does not exist (i.e., 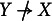 ). However, this independence may no longer hold when 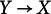 . This notion of “independence” can be formalized mathematically in at least three distinct ways: (1) through Kolmogorov complexity (Janzing & Schölkopf, 2010), (2) by examining the uncorrelatedness between *p* and the derivative of *f* (Janzing & Schölkopf, 2015), and (3) through the uncorrelatedness between *p* and the logarithm of the derivative of *f* (Daniusis et al., 2010). Whereas the mathematical formulation of Schölkopf et al.’s (2012) insight necessarily varies with this underlying notion of independence—e.g., see Janzing and Schölkopf (2015, Lemma 1) for one variation—the two observations discussed before hold true regardless of this specific mathematical formulation.

### 3.2 Mathematical analysis of cross-lagged effects

To mathematically illustrate the link between causality testing and semi-supervised learning, we use the CLPM (Hamaker et al., 2015) as an example of the underlying data-generating process. This analysis aims to emphasize two key observations. First, in the absence of reverse causality (i.e., 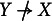 ), no information about the coefficient representing the 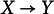 relationship can be inferred from a dataset containing only *X*. Second, when reverse causality exists (i.e., 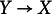 ), a standard least squares estimate for the coefficient representing 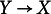 can be obtained even without *Y* in the dataset.

In its simplest form, CLPM assumes a model of structural equations: 
(1)

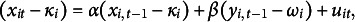



(2)

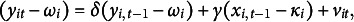

where 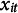 and 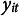 are the values of *X* and *Y* at time *t*, respectively; 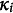 and 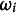 represent the sample-specific random intercepts for *X* and *Y*, respectively; and 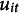 and 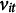 are i.i.d. random impulses. With this model, the cross-lagged parameter 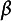 captures the (within-person) reverse causal effect 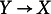 .

As the sample-specific random intercepts 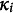 and 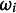 are extraneous to our ensuing analysis, we assume that 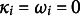 for all 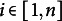 (where *n* is the sample size). For the ease of understanding, we also assume that all coefficients 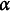 , 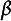 , 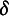 , and 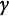 remain unchanged between time 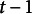 and 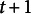 . These simplifying assumptions yield the following structural equations: 
(3)

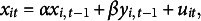



(4)

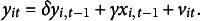

The crux of connecting semi-supervised learning with causality testing hinges on the following inquiry: Can any information about the regression coefficients in Equation 4 (i.e., 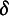 and 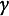 for predicting *Y*) be inferred from observations of *X* at time 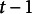 , *t*, and 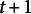 (i.e., 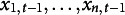 ; 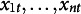 ; 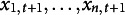 ), but no observation of *Y*, assuming the other coefficients (i.e., 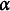 and 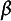 ) as given?

When 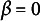 , this is obviously impossible because Equation 3 would only contain an autoregressive term 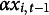 and the remainder 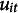 , meaning that we would have no information about *Y* from any of the inputs *X*, 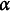 , and 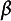 .

When 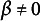 , however, knowledge of 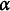 and 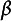 would allow us to make a probabilistic inference of 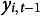 from Equation 3 based on 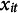 and 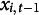 and, similarly, 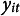 based on 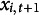 and 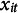 . Substituting these inferred values of 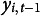 and 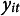 into Equation 4 would then let us make a probabilistic inference of the regression coefficients 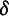 and 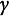 . To elucidate the reasoning behind this procedure, we rewrite Equation 3 as 
(5)

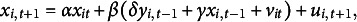



(6)

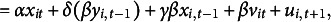



(7)

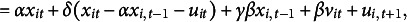

which can be further simplified to 
(8)

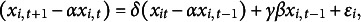

where 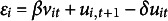 is a remainder term that aggregates various random impulses and satisfies 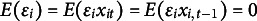 . Equation 8 can be used to obtain a standard least squares estimate for 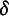 and 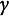 , i.e., 
(9)

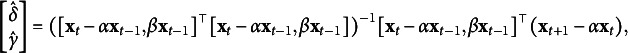

where 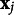 represents the vector of *X* at time *j*. The variances of 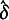 and 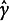 are 
(10)

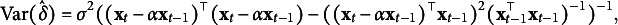



(11)

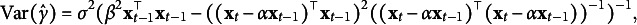

where 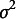 is the variance of 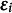 . Note from Equation 11 that the variance of 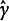 tends to infinity when 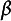 approaches 0. This substantiates our prior assertion that estimating the coefficient for 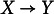 (i.e., 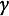 ) from *X* is only feasible when the coefficient for 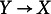 (i.e., 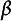 ) is significant.

Given that 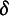 and 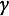 can be inferred (at least asymptotically) using just three inputs— 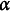 , 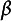 , and *X*—without any information on *Y*, a natural question is: which among the three inputs carries information about 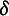 and 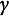 ? We can rule out 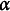 and 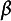 because they are free parameters (independent of 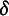 and 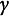 ) per CLPM. This leaves the only possibility to be that the distribution of *X* carries certain information about the 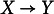 relationship (i.e., 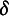 and 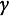 ). Importantly, this information carriage is valid if and only if reverse causality 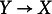 is present (i.e., 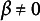 ).

At this juncture, the connection between semi-supervised learning and reverse causality testing becomes evident. Recall that a semi-supervised learning algorithm aims to learn the 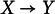 relationship 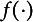 —i.e., 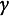 in the case of CLPM—using a small labeled set of 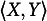 pairs and a large unlabeled set consisting solely of *X*. Clearly, the only information revealed by the unlabeled set is the distribution of *X*. When reverse causality does not exist (i.e., 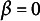 ), the distribution of *X* does not carry any information about 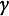 . In this case, the unlabeled set is useless for improving the prediction of *Y*, making the deployment of a semi-supervised learning algorithm futile. However, when reverse causality exists (i.e., 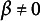 ), the distribution of *X*—and therefore the unlabeled set—does carry information about 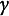 (as shown in Equation 9), making semi-supervised learning potentially fruitful.

For a more specific example, consider the aforementioned self-training method for semi-supervised learning. When the *X*–*Y* relationship is unidirectional flowing from *X* to *Y* (i.e., 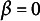 ), expanding the labeled set with pseudo-labels will not lead to a more accurate prediction of *Y* for the simple reason that even an infinitely large unlabeled set, which perfectly reveals 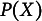 , still contains no information about the 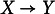 relationship. Put simply, we can test reverse causality by comparing the predictive accuracy pre and post self-training, with an increase in predictive accuracy suggesting the existence of reverse causality.

### 3.3 Generalized test

Whereas we developed the connection between semi-supervised learning and reverse causality testing through the structural model of CLPM, the connection indeed persists regardless of the underlying data-generating process. To understand why, consider an extension of Equations 3 and 4 to data-generating processes beyond CLPM. When the *X*–*Y* relationship is unidirectional flowing from *X* to *Y*, we have 
(12)

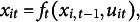



(13)

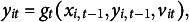

where 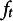 and 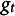 can be any arbitrary stochastic function.

Two key insights emerge from the equations. First, the 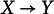 relationship is wholly captured by 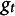 . Second, alterations in 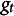 has no influence on the value (and distribution) of *X*. Combining these two insights, it is evident that the distribution of *X* does not carry any information about the 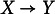 relationship unless there exists a reciprocal 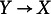 relationship. This underscores that the nexus between semi-supervised learning and reverse causality testing remains intact, independent of assumptions about the data-generating process.

## 4 Reverse causality testing using semi-supervised learning

In this section, we develop our novel method for reverse causality testing. As established in the last section, a semi-supervised learning algorithm can effectively leverage the large unlabeled (i.e., *X*-only) data to improve predictive accuracy for *Y* only when reverse causality 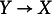 is at play. Building upon this insight, our method revolves around assessing the predictive accuracy of semi-supervised learning. In the passages that follow, we outline our methodological design in two steps. First, we describe the input and output of semi-supervised learning, explaining in detail how the output of semi-supervised learning is used for reverse causality testing. Then, we delve into the algorithmic design of semi-supervised learning.

### 4.1 Input and output of semi-supervised learning

The objective of our method is to detect the presence of reverse causality, i.e., 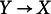 , given a longitudinal panel dataset 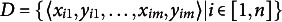 , where *n* is the sample size and *m* is the number of waves. Our method requires at least three waves (i.e., 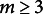 ) for identification. It imposes no assumptions about the lag, provided that the lag is not so extensive that 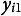 exerts no causal effect on 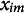 , as this would render reverse causality unidentifiable within the given dataset. Our method also makes no assumption about the functional form of the relationships between *X* and *Y*. The sole assumption underlying our method is the working condition of causal learning, which states that 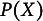 is independent^4^ of *f* if 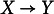 fully describes the relationship between *X* and *Y* (Schölkopf et al., 2012). Also note that, if the purpose is to test the existence of 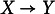 instead, the only revision required is to swap *X* and *Y* in the input data.

#### 4.1.1 Input

Recall from the literature review that a semi-supervised learning algorithm takes as input two datasets, a small labeled set 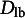 , which consists of 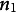 data points with paired *X*–*Y* values, and a large unlabeled set 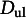 , which consists of 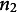 data points ( 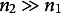 ) with *X* values only. To properly specify 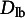 and 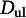 based on the input data *D*, there are three issues to be addressed.

First, since semi-supervised learning algorithms generally require the prediction target *Y* to be a scalar (i.e., a single variable), we need to select (the *Y* value of) one wave as the prediction target for semi-supervised learning. Mathematically, any wave except the last one would work because, as discussed earlier, if 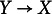 exists with a lagged effect, then the distribution of *X* in the *t*th wave carries information about *Y* in all previous waves (i.e., 1 to 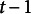 ). In other words, semi-supervised learning could effectively boost the predictive accuracy toward *Y* for all but the last wave. Since we are interested in minimizing the number of waves required for identification, a proper choice is to select *Y* in the first wave, i.e., 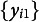 , as the prediction target because otherwise data in the first wave would become useless for identification (as *Y* in a latter wave cannot have a causal effect on *X* in the first wave).

Second, we need to determine the variable composition of *X*. When 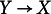 exists, the distribution of *X* from the second wave onward is informative for predicting the value of *Y* from the first wave. We therefore include 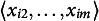 as the predictor vector *X*.

Third, we also need to determine 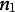 and 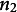 , the sample sizes for 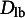 and 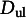 , respectively. Recall that our purpose is to test whether semi-supervised learning can effectively leverage the unlabeled set 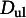 to enhance predictive accuracy. Clearly, assuming 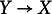 exists, the smaller 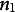 and the larger 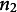 is, the more likely we would be able to detect the effectiveness of semi-supervised learning. From this perspective, a natural choice is to make 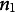 the minimum labeled-sample size required by the semi-supervised learning algorithm, and to include all other data in the unlabeled set (i.e., 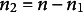 ). For example, the aforementioned self-training algorithm generally requires 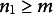 to avoid an initial degenerate solution. Hence, we generate the labeled set 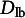 and the unlabeled set 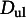 by first randomly permuting the order of all data points in *D*, before setting 
(14)

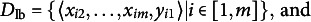



(15)

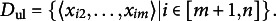



#### 4.1.2 Output

Our method for reverse causality testing focuses on the accuracy of 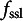 , the output of semi-supervised learning. To determine whether the unlabeled set 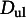 is useful for improving the predictive accuracy of 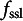 (which is only possible when reverse causality exists), we compare 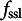 against a baseline model 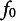 , which is the initial model of semi-supervised learning generated from the small labeled set 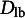 only *before* accessing the unlabeled set 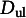 . A smaller error of 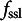 would serve as evidence against the null hypothesis of no reverse causality.

Recall from earlier discussions that 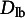 and 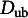 are drawn uniformly at random from the input data *D*. Since 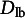 and 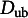 are random samples, comparing the predictive errors of the machine learning models trained on them—i.e., 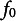 and 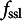 , respectively—requires a statistical significance test. Specifically, we need to determine whether any observed reduction in predictive error of 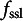 relative to 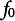 is statistically significant. To assess this, we repeatedly draw i.i.d. samples of 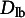 from *D*, derive the corresponding 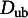 , and compare the predictive error of 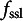 against 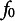 for each sample. The statistical significance of the reduction in predictive error can then be evaluated based on these comparisons. There are three key issues worth discussing in this design: (1) how to measure the predictive error of a model, (2) how to assess the statistical significance of the reduction in predictive error, and (3) how to determine the number of resamples necessary for the test.

First, in terms of measuring the predictive error of a machine learning model, a prevalent practice in machine learning is to separate the testing dataset from the training data due to concerns of overfitting (Bishop & Nasrabadi, 2006). It is important to note that overfitting is *not* a concern in our case because the prediction target 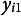 for the vast majority of data (i.e., 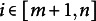 ) is *hidden* from semi-supervised learning. As such, we can assess the predictive error of 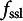 (and the baseline 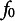 ) directly over the input dataset *D*. Further, our method has no specific requirement on the metric for predictive error, as a reduction of any error metric indicates the existence of reverse causality. For example, when 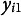 is binary, we could use the total number of prediction errors as the metric. The cross-entropy metric (Bishop & Nasrabadi, 2006) can be used for categorical 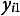 . When 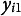 is continuous, the error metric could be the mean squared error, mean absolute error, etc.

Second, for comparing the predictive errors of two machine learning models, Demšar (2006) reviewed three types of statistical tests used in the literature: (1) a parametric test, like the paired *t*-test (Dietterich, 1998), (2) a nonparametric test that makes assumptions about the distribution of difference in predictive accuracy, like the Wilcoxon signed-rank test (Santafe et al., 2015), and (3) a nonparametric test that only requires predictive accuracy to be comparable (i.e., at least on an ordinal scale), like the binomial test, also known as the sign test (Salzberg, 1997).

Problems with parametric tests such as the paired *t*-test have been well documented in the literature (Dietterich, 1998). As summarized by Demšar (2006), these tests suffer from sensitivity to outliers and the issue of commensurability between different runs, making it possible for the total failure of semi-supervised learning on a single sample of 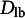 to dominate the test result. Similarly, key assumptions of the Wilcoxon signed-rank test do not hold in our context. For instance, it assumes that the difference in predictive accuracy between the two models is *symmetrically* distributed around a central value, yet there is no evidence to support this symmetry in our setting. In fact, semi-supervised learning is likely to amplify the skewness in the labeled set, suggesting that the difference could follow a heavy-tailed rather than symmetric distribution, violating the assumptions of the Wilcoxon signed-rank test. This leaves the (exact) binomial test (i.e., sign test) as a suitable alternative, as it requires only that each pair of predictive accuracy scores be comparable (Salzberg, 1997) and is inherently robust to outliers (Demšar, 2006). In our case, we employ the binomial test (with 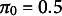 ) to compare the number of runs (i.e., 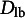 samples) where 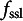 outperforms 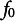 against the number of runs where the reverse is true.

Finally, two key factors influence the determination of the number of resamples required for the test. First, increasing the number of resamples enhances the statistical power of the test, which is especially critical given the well-documented limitation of the binomial test in terms of low statistical power (Demšar, 2006; Rainio et al., 2024; Salzberg, 1997). Second, a larger number of resamples incurs higher computational costs, as the semi-supervised learning algorithm must be executed for each sampled 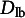 and 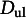 . This computational burden can be particularly significant for resource-intensive algorithms, such as those based on deep learning (Goodfellow et al., 2016). To balance these considerations, we recommend following the default setting proposed by Demšar (2006) and performing 1,000 resamples. As demonstrated in the simulation studies, this setting offers a practical trade-off between statistical power and computational efficiency.

### 4.2 Design of semi-supervised learning algorithm

Numerous algorithms have been proposed for semi-supervised learning (Van Engelen & Hoos, 2020). We chose to implement the self-training paradigm discussed earlier. This choice is driven by two primary considerations. First, self-training is highly versatile, as it can be seamlessly integrated with a wide range of supervised learning algorithms as its base learner (Sohn et al., 2020). Second, the mathematical analysis of self-training is one of the few that have been thoroughly developed in the literature (Amini & Gallinari, 2002; Grandvalet & Bengio, 2004), providing a clear connection between the Bayes risk of its predictions and the foundational working condition of semi-supervised learning that has been discussed earlier in the article.

In the passages that follow, we describe the mathematical foundation and algorithmic design of self-training. Note that, our method represents a novel use of self-training for the purpose of reverse causality testing, and we did not make any change to its canonical algorithmic design. For the ease of discussion, we start with a simple setting where the prediction target 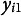 is binary (i.e., 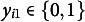 ) and the underlying learning algorithm is logistic regression. At the end of this section, we describe a generalization to continuous variables and any regression algorithm.

#### 4.2.1 Mathematical foundation of self-training

Any algorithm aiming to learn a prediction model that estimates a binary 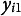 based on a predictor vector 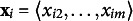 can be viewed as learning a function^5^ 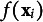 that approximates 
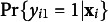
 . For example, logistic regression specifies 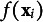 as 
(16)

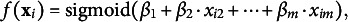

where 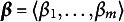 are the coefficients to be estimated from training data.

Semi-supervised learning in general, and self-training in particular, starts by estimating 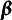 from the small labeled set 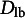 . Specifically, it does so by finding 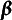 that maximizes the following log-likelihood function: 
(17)

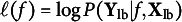



(18)

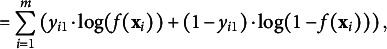

where 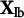 and 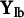 represent the predictor (i.e., 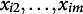 ) and prediction target (i.e., 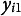 ) portions of 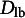 , respectively. In the Bayesian framework, these initial coefficient estimates form the maximum a posteriori (MAP) estimate under the uniform prior. As shown by Seeger (2000), this MAP estimate does *not* change if we merely add the unlabeled set 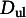 into the observed data, because semi-supervised learning only works under the condition that the distribution of 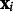 reveals information about 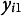 . In other words, in order to proceed beyond this initial step and generate the MAP estimate for semi-supervised learning, we need to encode its working condition into the prior distribution used to calculate the MAP estimate.

In the Bayesian framework, a common method for deriving the prior distribution from a given constraint (e.g., the working condition for semi-supervised learning) is the principle of maximum entropy (Jaynes, 1957), which sets the prior distribution as the one that satisfies the given constraint while having the maximum information entropy (Cover & Thomas, 2006). Grandvalet & Bengio (2004) followed this principle to prove that, for a semi-supervised learning algorithm that uses both 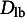 and 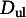 , the MAP estimate for 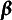 is the maximizer of the following criterion 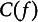 , 
(19)

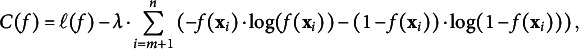

where 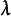 ( 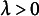 ) is the Lagrange multiplier that, roughly speaking, captures the amount of information about 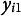 that can be revealed by the distribution of 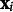 . Amini & Gallinari, 2002 analyzed self-training with logistic regression as the underlying learning algorithm, and proved that the resulting coefficient estimates maximizes 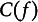 when 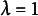 . In other words, semi-supervised learning in general, and self-training in particular, can be viewed as approximating the MAP estimate of *f* under the working condition of semi-supervised learning. Specifically, it does so by finding *f* that maximizes 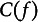 in Equation 19.

#### 4.2.2 Algorithmic design of self-training

The algorithmic design of self-training can be readily derived from Equation 19. Note that the second term in the equation is proportional to the sum of 
(20)

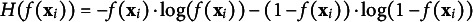

for all data points in the unlabeled set 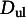 . Recall that 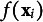 is the prediction from semi-supervised learning for 
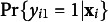
 . This makes 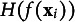 in Equation 20 the entropy (Cover & Thomas, 2006) of the predicted distribution of 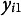 given 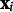 , meaning that it captures the amount of *uncertainty* in machine learning predictions. For example, we have 
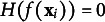
 , its minimum possible value, when semi-supervised learning is fully confident of its prediction (i.e., 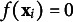 or 1). In contrast, when 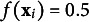 (i.e., maximum uncertainty), 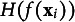 reaches its maximum value^6^ of 1.

With this understanding, the goal of self-training—i.e., the maximization of 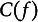 —pertaining to the unlabeled set is equivalent with minimizing prediction uncertainty (i.e., 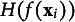 ) for the unlabeled data. Self-training does so in an iterative manner. As described earlier, the initial iteration estimates 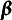 from the labeled set 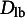 by applying the underlying learning algorithm, which in this case is the standard logistic regression. The estimated 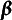 is then used to compute 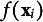 , and thereby 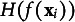 , for all data points in the unlabeled set 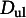 . The first iteration concludes by adding into the labeled set 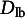 all unlabeled data points with prediction uncertainty below a pre-determined threshold *h*, using their predicted labels as if they were real. In other words, the labeled and unlabeled sets are updated as 
(21)

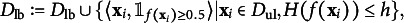



(22)

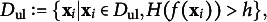

where 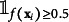 is the predicted label for 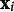 —i.e., an indicator function that returns 1 if 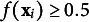 and 0 otherwise. The updated 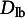 and 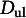 are then entered as input to the next iteration. The iterative process ends when no new *X*–*Y* pair is added to 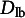 after an iteration. As can be seen from the description, self-training minimizes prediction uncertainty among unlabeled data using the idea of *pseudo-labels*, i.e., by promoting those with minimal prediction uncertainty to the labeled set, using their predicted labels as if they were real. These promoted data points, in turn, reduce prediction uncertainty for the remaining unlabeled data (Amini & Gallinari, 2002), pushing the coefficient estimates closer to their MAP values that maximize 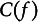 .

#### 4.2.3 Generalization to continuous variables

Whereas the above description of self-training is based on a binary 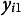 and logistic regression being the underlying learning algorithm, the same iterative process can be adapted to support continuous 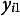 and any underlying learning algorithm. This adaption requires addressing two issues. One is the design of an appropriate uncertainty measure (i.e., 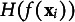 in the binary case), and the other is the generation of pseudo-label (i.e., 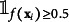 in the binary case). The reason why these two issues arise for continuous variables is because, unlike in the binary case where 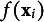 , as an estimate of 
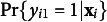
 , inherently captures uncertainty about the predicted 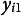 , a point-estimate for a continuous 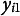 contains no such uncertainty information. Therefore, instead of deriving both the uncertainty measure and the pseudo-label from 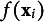 itself, we may have to resort to other information in order to do so in the continuous case.

A well-known method for addressing both issues in semi-supervised learning is co-training (Blum & Mitchell, 1998). With this method, instead of generating a single prediction of 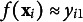 , we generate two predictions, 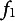 and 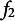 , based on two (slightly) different subsets of variables in 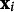 . Then, the difference between 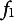 and 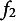 (i.e., 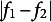 ) is a natural measure of uncertainty, while the mean of the two (i.e., 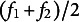 ) can be used as pseudo-label for expanding the labeled set.

More specifically, recall that our method includes in 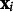 a total of 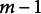 variables 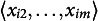 , where 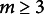 because we require a minimum of three waves for identification. A natural choice is to associate 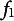 with the first 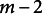 variables 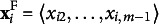 , and 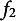 with the last 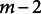 variables 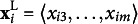 . This way, we allow a divergence of two predictions (to allow the uncertainty estimate) while minimizing the number of predictors withheld from either. With this design, the updates of labeled and unlabeled set in each iteration become 
(23)

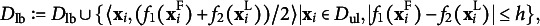



(24)

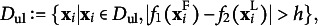

whereas everything else in the iterative process follows directly from the binary case. Clearly, this design for continuous 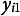 is compatible with any underlying learning algorithm for generating 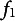 and 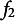 , e.g., linear regression (Stine, 1985), support vector machines (SVMs; De Brabanter et al., 2010), neural networks (Heskes, 1996), etc. The pseudocode of our algorithm for continuous variables is available in Table 1.

**Table 1:**
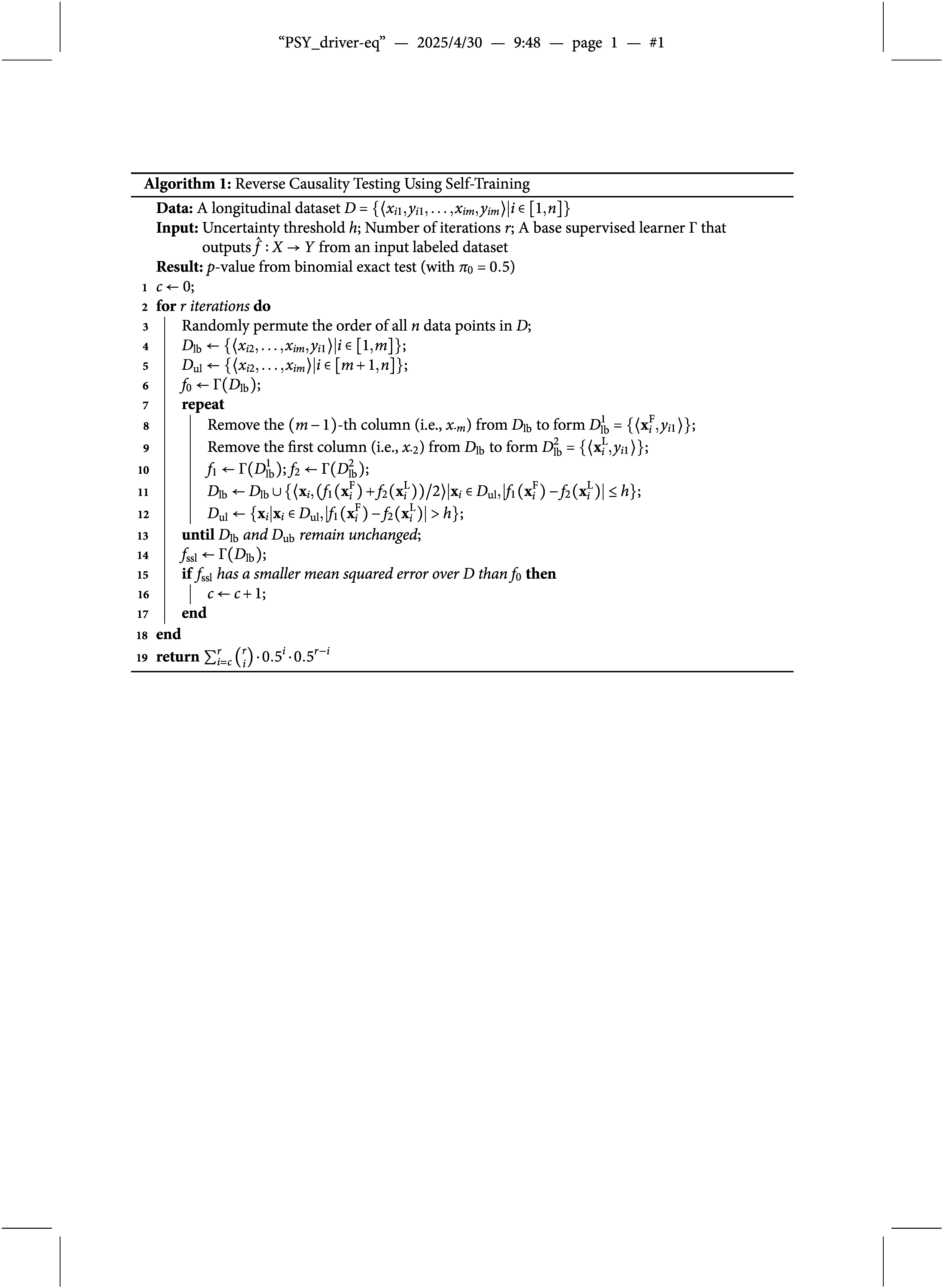
Pseudocode for reverse causality testing with continuous variables

### 4.3 Transparency and openness

The complete code implementation of our algorithm is publicly available at https://github.com/calearn/revc (Python) and https://github.com/calearn/revc_m (MATLAB).

## 5 Simulation studies

We conducted two simulation studies. The main study evaluates the statistical power of our method in identifying reverse causality, and a followup study assesses the Type I error rate of our method in the absence of reverse causality, because existing methods such as the random-intercept CLPM (RI-CLPM; Hamaker et al., 2015) are known to generate Type I errors.

### 5.1 Data-generating process

In the main simulation study, we aimed to delineate the primary factors influencing the statistical power of our method. To achieve this, we employed a straightforward structural model-Equations 1 and 2—as the data-generating process, establishing a clear ground truth for the causal direction. To ensure a comprehensive analysis, we created multiple levels for three key parameters in the data-generating process: the total number of observations *N* and the cross-lagged parameters 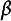 (i.e., 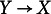 ) and 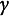 (i.e., 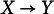 ). For the sample size *N*, we created four levels: 100, 250, 500, and 1,000. For the cross-lagged parameters 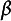 and 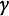 , we created six levels for each: 0, 0.1, 0.2, 0.3, 0.4, and 0.5. In total, the design for the main simulation study consists of 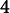 (*N*) 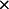 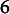 ( 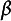 ) 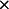 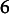 ( 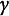 ) = 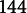 unique conditions.

We adopted a standard parameter setup (e.g., Hamaker et al., 2015) to configure the other fixed parameters for the data-generating process in the main simulation study. Specifically, we followed Hamaker et al. (2015) to set the autoregressive parameters for both variables to 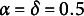 . All random impulses 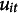 and 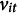 were generated from a Gaussian distribution 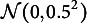 , while the sample-specific random intercepts were fixed at 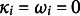 for all 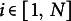 . To generate the initial (Wave 1) values of 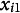 and 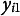 , we deliberately used different distributions^7^ to emphasize that our method does not rely on specific distributional assumptions for the input data. For each 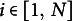 , 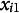 was sampled uniformly at random from the interval 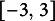 , while 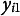 was drawn from a Gaussian distribution 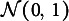 .

In the followup simulation study, we followed Lucas (2023), which demonstrates the spurious cross-lagged effects generated by CLPM, in adopting the widely used Stable Trait Autoregressive Trait and State (STARTS) model (Kenny & Zautra, 1995) as the underlying data-generating process. We also followed Lucas (2023) in the parameter setup, specifically by setting (for both *X* and *Y*) the stability parameter as 0.5, the random intercept variance as 1, and variance of autoregressive component as 1. We also added a measurement error of variance 0.3 to both *X* and *Y*. Since the focus of the followup study is on Type I errors, the cross-lagged parameter for reverse causality ( 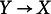 ) was always set to zero, while the parameter for 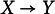 (represented as 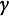 for consistency with the main study) was varied from 0.1 to 0.5. Like in the main study, we created four levels, 100, 250, 500, and 1,000, for the sample size *N*. In total, the design for the followup simulation study consists of 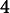 (*N*) 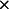 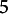 ( 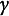 ) = 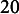 unique conditions.

### 5.2 Algorithmic implementations

Recall from earlier discussions that our method applies to both discrete and continuous *Y*, and can be used with any supervised learning algorithm as its base learner. To demonstrate the versatility of our method, we implemented two variants of it. The first was designed for continuous *Y* and implemented in MATLAB R2023b (with statistics and machine learning toolbox). We selected a simple design, i.e., OLS regression, as the underlying learning algorithm. We set the number of resamples for binomial test to be 1,000, leading to a computational overhead of about 20 seconds per simulation run on a laptop computer with Apple M2 CPU and 8GB RAM. For the number of waves taken as input, we set 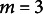 (i.e., the first three waves), the minimum value that satisfies the identification requirement of our method while providing a conservative estimate of its statistical power. For the uncertainty threshold *h* (i.e., the maximum difference in prediction between the two models for an unlabeled data point to be added to the labeled set, see Equation 24), after testing a wide range of threshold values, we found that the output of our method is insensitive to the threshold setting as long as it is neither too large—so as to allow all unlabeled data to enter the labeled set at once—nor too small to permit any unlabeled data point into the labeled set. Due to this finding, we set the threshold to a constant of 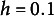 , which is about 10% of the standard deviation of the label (i.e., *Y*).

Since the OLS regression algorithm relies on a linear model and our data-generating process is also linear, concerns may arise regarding a potential unfair advantage due to their inherent consistency. To address these concerns, we implemented a variant of our method using a nonlinear learning model. Specifically, for the underlying base learner in self-training, we employed the SVM (Cortes & Vapnik, 1995) algorithm with a polynomial kernel of degree 3 (i.e., a cubic kernel SVM).

Given the significantly higher computational overhead of SVM, we implemented this variant in Python using the scikit-learn (Pedregosa et al., 2011) machine learning library. We adhered to the default settings of scikit-learn’s LIBSVM implementation (Chang & Lin, 2011) for all SVM parameters, including the kernel configuration (i.e., in function sklearn.svm.SVC). For the uncertainty threshold in the self-training algorithm, we used the built-in options for the criterion parameter in sklearn.semi_supervised.SelfTrainingClassifier. Apart from the use of SVM as the base learner, all other design elements were identical to the OLS implementation, except for an additional input data normalization process required for the SVM implementation. Specifically, *Y* was converted to binary values (using median dichotomization), and *X* was normalized to the range 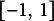 (through min-max scaling) to account for SVM’s sensitivity to input feature scaling (Chang & Lin, 2011; Tax & Duin, 2004).

To accommodate the computational demands of the nonlinear SVM algorithm and the large number of simulation runs required for the main study, this implementation was executed on Amazon Web Services (AWS) Batch using Elastic Container Service (ECS) clusters configured with 256 virtual CPUs, provisioned and scaled automatically using AWS Fargate. With the same number of resamples (1,000) as the OLS implementation, the SVM variant completed each simulation run in approximately 30 seconds on the AWS cluster.

### 5.3 Simulation results

For the main study, Table 2 presents the statistical power achieved by our method under a significance level of 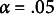 for all simulation settings with 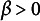 . For settings where 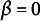 (highlighted in gray), the table reports the Type I error rates. Each cell in the table is based on 1,000 independent runs of our method. Note that results for the nonlinear SVM-based implementation are included only for 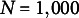 , as the SVM-based implementation required more than 500 samples to consistently converge to reliable predictions.

**Table 2:** Type I error rate and statistical power of our method in the main simulation study

		OLS				SVM
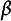	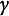	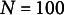	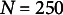	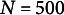	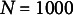	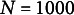
0.0	0.0	0.005	0.003	0.037	0.000	0.017
0.1	0.0	0.006	0.188	0.335	0.448	0.518
0.2	0.0	0.091	0.301	0.976	0.972	1.000
0.3	0.0	0.440	0.774	0.998	1.000	1.000
0.4	0.0	0.593	0.999	0.999	1.000	1.000
0.5	0.0	0.820	1.000	1.000	1.000	1.000
0.0	0.1	0.001	0.058	0.002	0.000	0.032
0.1	0.1	0.000	0.018	0.557	0.421	0.542
0.2	0.1	0.092	0.636	0.800	0.719	1.000
0.3	0.1	0.362	0.589	0.988	0.986	1.000
0.4	0.1	0.503	0.994	0.998	1.000	1.000
0.5	0.1	0.856	0.908	1.000	0.999	0.990
0.0	0.2	0.073	0.000	0.013	0.000	0.018
0.1	0.2	0.012	0.073	0.469	0.886	0.546
0.2	0.2	0.202	0.669	0.763	0.982	1.000
0.3	0.2	0.373	0.793	0.833	0.997	1.000
0.4	0.2	0.618	0.990	0.995	1.000	0.986
0.5	0.2	0.731	0.943	0.935	0.992	0.841
0.0	0.3	0.050	0.000	0.071	0.007	0.036
0.1	0.3	0.004	0.179	0.702	0.575	0.487
0.2	0.3	0.157	0.311	0.586	0.816	1.000
0.3	0.3	0.306	0.816	0.999	0.961	0.997
0.4	0.3	0.373	0.855	0.979	0.998	0.909
0.5	0.3	0.309	0.879	0.914	0.975	0.510
0.0	0.4	0.000	0.004	0.001	0.037	0.048
0.1	0.4	0.008	0.052	0.323	0.475	0.456
0.2	0.4	0.277	0.320	0.688	0.933	1.000
0.3	0.4	0.446	0.615	0.896	0.939	0.993
0.4	0.4	0.401	0.726	0.882	0.863	0.725
0.5	0.4	0.475	0.575	0.838	0.792	0.370
0.0	0.5	0.058	0.057	0.040	0.001	0.037
0.1	0.5	0.003	0.018	0.144	0.343	0.504
0.2	0.5	0.255	0.429	0.648	0.740	0.997
0.3	0.5	0.240	0.501	0.931	0.930	0.966
0.4	0.5	0.243	0.486	0.704	0.879	0.668
0.5	0.5	0.590	0.347	0.840	0.856	0.432

We can draw three key observations from the table. First, regarding Type I error rates (highlighted rows with 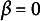 ), our method consistently remains below 0.075 across all simulation conditions, aligning with Bradley’s (1978) liberal robustness criterion. This demonstrates the robustness of our method against finding spurious reverse causal effects.

Second, note that the top six rows of Table 2, along with the highlighted rows, represent simulation conditions where *X* and *Y* exhibit a unidirectional relationship. The statistical power achieved by our method in these scenarios highlights its effectiveness in identifying the direction of a unidirectional effect—a critical use-case for our method when competing theories stipulate different causal directions between *X* and *Y*. For example, Rows 3–6 show that, when 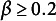 , the OLS implementation of our method achieves statistical power exceeding 0.97 when 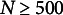 , while the SVM implementation achieves the same when 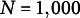 .

Third, the statistical power of our method is influenced by three key factors. One factor is *N*, the input sample size. Larger sample sizes significantly improve statistical power. For example, when 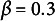 and 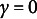 , the OLS implementation achieves a statistical power of only 0.44 with 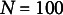 but exceeds 0.99 when 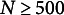 . Another factor is 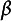 , the cross-lagged parameter representing the strength of the reverse causal effect. When 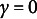 (i.e., unidirectional effect), larger 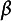 values correspond to higher statistical power. For instance, with 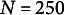 and 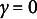 , the OLS implementation achieves a power of 0.19 at 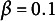 , increasing to 0.77 at 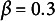 , and exceeding 0.99 for 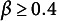 . The SVM implementation exhibits a similar trend. The final factor is 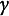 , the strength of the causal effect 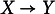 . The impact of 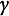 on statistical power varies depending on other parameters. For example, when 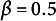 and 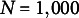 , a stronger forward effect of 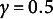 can obscure reverse causality, reducing power from 1.00 at 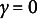 to 0.86 at 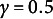 . Conversely, when 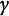 and 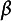 are smaller yet closer in magnitude (e.g., 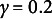 , 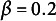 , 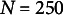 ), 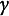 could potentially enhance power, increasing it from 0.30 at 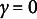 to 0.67 at 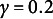 . This increase can be attributed to the technical design of the two-step self-training algorithm in our method. Specifically, in situations where the causal effect 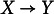 is nonexistent (i.e., 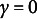 ), the initial step of self-training, which learns from the labeled set only, is destined to yield highly inaccurate predictions. In such scenarios, particularly with smaller sample sizes, it becomes challenging for the subsequent step (of learning from the unlabeled set) to significantly enhance predictive accuracy even in the presence of reverse causality. Therefore, a larger 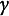 value, which improves the accuracy of the initial step, also tends to boost the statistical power.

Interestingly, the negative impact of 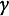 on the statistical power of our method appears more pronounced in the SVM implementation compared to the OLS implementation. For example, with 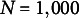 , the OLS implementation maintains a statistical power of at least 0.70 for 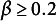 , regardless of 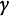 . In contrast, the SVM implementation’s power drops from 1.00 at 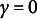 to 0.43 at 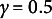 for 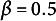 . We attribute this discrepancy to SVM’s sensitivity to feature scaling (Tax & Duin, 2004) and vulnerability to outliers (Debruyne, 2009), both of which are exacerbated at larger 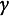 .

Table 3 summarizes the results of the follow-up simulation study comparing the Type I error rates of RI-CLPM and the OLS implementation of our method.^8^ Following Mulder & Hamaker (2021), RI-CLPM was implemented using default settings in Mplus.^9^ The results reveal that RI-CLPM frequently identifies spurious reverse causal relationships, with Type I error rates reaching 0.88 when 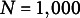 and 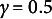 . In general, RI-CLPM’s Type I error rate increases with larger *N* and higher 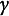 . In contrast, our method maintains a Type I error rate below 0.05 across all conditions, again aligning with Bradley’s (1978) liberal robustness criterion and demonstrating an advantage of our method over CLPM and its variants, which are known to find spurious cross-lagged effects (Lucas, 2023).

**Table 3:** Type I error rate of RI-CLPM and our method under STARTS model

		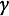 = 0.1	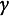 = 0.2	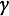 = 0.3	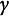 = 0.4	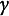 = 0.5
RI-CLPM	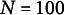	0.055	0.077	0.099	0.139	0.186
	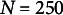	0.066	0.096	0.174	0.262	0.352
	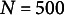	0.073	0.148	0.277	0.445	0.614
	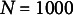	0.106	0.254	0.478	0.713	0.883
Our Method	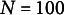	0.001	0.002	0.012	0.006	0.002
	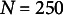	0.001	0.000	0.004	0.004	0.013
	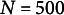	0.022	0.055	0.038	0.022	0.018
	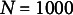	0.003	0.015	0.012	0.007	0.016

## 6 A case study

We applied our method over a real-world panel dataset to demonstrate its value for reverse causality testing in practice. Specifically, we examined the relationship between work-family conflict (WFC)—i.e., “a form of inter-role conflict in which the role pressures from the work and family domains are mutually incompatible in some respect” (Greenhaus & Beutell, 1985, p. 77)—and job satisfaction (JAS)—i.e., the “overall evaluative judgement one has about one’s job” (Judge et al., 2017, p. 357). We chose this focal relationship for two main reasons. First, like many constructs in industrial-organizational psychology, it is often practically difficult, if not ethically dubious, to manipulate WFC or JAS in a randomized controlled trial. Second, whereas a robust correlation between WFC and JAS has been widely recognized (Allen et al., 2020; Amstad et al., 2011), there are ongoing debates about the causal direction between the two constructs. Some posit a unidirectional effect of WFC negatively affecting JAS (e.g., Allen et al., 2020; Amstad et al., 2011; Kossek & Ozeki, 1998). Others contend that the causal influence flows in the opposite direction, as higher JAS leads to greater work-life balance and, correspondingly, lower WFC (Landolfi et al., 2022). Yet others suggest the existence of reciprocal effects, with WFC and JAS affecting each other over time (e.g., Demerouti et al., 2004). Given these varied viewpoints, we sought to apply our method to empirically test the existence of causal effect in either direction.

### 6.1 Data

We drew from the Swiss Household Panel (SHP Group, 2023; Voorpostel et al., 2023) to examine the WFC–JAS relationship. The SHP began in 1999 with a nationally representative sample of 5,074 Swiss Households, introducing supplementary samples in 2004 (addition of 2,537 households), 2013 (addition of 3,989 households), and 2020 (addition of 4,380 households; Voorpostel et al., 2023). All household members aged 14 and above are surveyed annually via telephone and written surveys. We mirrored the timeframe used in prior work on CLPM and analyzed data collected in Waves 6 through 9 (i.e., the annual surveys between 2004 and 2007; Ozkok et al., 2022). We excluded participants who did not work or who did not complete a single survey in our timeframe, resulting in *N* = 7,748. Participants (51.55% women, 48.45% men) were on average 39.28 (SD = 14.50) years old. On average, participants had 12.98 (SD = 3.20) years of education.

In the case study, WFC was measured by a single item on a 11-point Likert scale (0 = not at all, 10 = very strongly). The item read “How strongly does your work interfere with your private activities and family obligations more than you would want this to be?” JAS was measured by a six-item measure created for the SHP on a 11-point Likert scale (0 = not at all satisfied, 10 = completely satisfied). The lead-in to the items was “Can you indicate your degree of satisfaction for each of the following points?” Sample items include “your job in general” and “the amount of your work.” The internal consistency for the four waves was .78, .78, .79, and .79, respectively.

### 6.2 Results

In terms of algorithmic implementations, we used the same implementations (OLS and SVM) as the simulation study, and set the number of resamples to 10,000 and 100, respectively. Note that we intentionally set the number of resamples for the OLS implementation to be much higher than required in order to support a detailed analysis described later. With either implementation, we first tested the presence of WFC 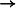 JAS by setting the self-training algorithm to predict WFC from JAS, before testing the presence of JAS 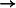 WFC by setting the self-training algorithm to predict JAS from WFC. For the OLS implementation, self-training was effective for predicting WFC from JAS in 5,743 out of 10,000 resamples (one-tailed binomial exact test with 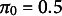 : 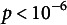 ), and was effective for predicting JAS from WFC in 5,006 out of 10,000 resamples (one-tailed binomial exact test with 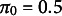 : 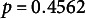 ). For the nonlinear SVM implementation, the results were consistent with the OLS implementation. Specifically, for the prediction of WFC from JAS, self-training was effective in 67 out of 100 iterations, leading to a *p*-value of 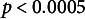 . On the flip side, when predicting JAS from WFC, self-training was effective only in 37 out of 100 runs,^10^ leading to a *p*-value of 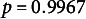 . In sum, the results suggest that work-family conflict influences JAS, but not vice versa.

To further inspect *why* semi-supervised learning succeed in predicting WFC but not JAS, we examined how its effectiveness depends on the small labeled set, which is the only information source about *Y* that semi-supervised learning receives. Recall from the design of self-training that, if the distribution of *X* reveals signals about the *X*–*Y* relationship, we would expect self-training to be *more* effective when the labeled set is a more representative sample of the full dataset. This is because, when the labeled set is a severely biased sample, say featuring only a single value of *Y*, then self-training would stand no chance in becoming effective, as it would not even know what the other values of *Y* might be.

To inspect how the effectiveness of semi-supervised learning varies with the bias of labeled sample, we leveraged the 10,000 resample runs executed for the OLS implementation. Specifically, we sorted all runs according to the distributional distance (as measured by Kolmogorov–Smirnov test statistic; Daniel, 1990) between the labeled set and the full dataset, before stratifying them into 10 equi-sized bins (i.e., each containing 1,000 runs) according to the sorted order and calculating the average effectiveness of semi-supervised learning for each bin as measured by the fraction of runs that improve predictive accuracy minus the fraction of those that reduce accuracy. As discussed before, if self-training were ineffective, we would expect the average effectiveness measure to hover around zero for all ten bins. In contrast, if self-training were effective, we would expect the average effectiveness to be high when the distributional distance is small, and gradually decrease when the distributional distance becomes larger.

**Figure 2:**
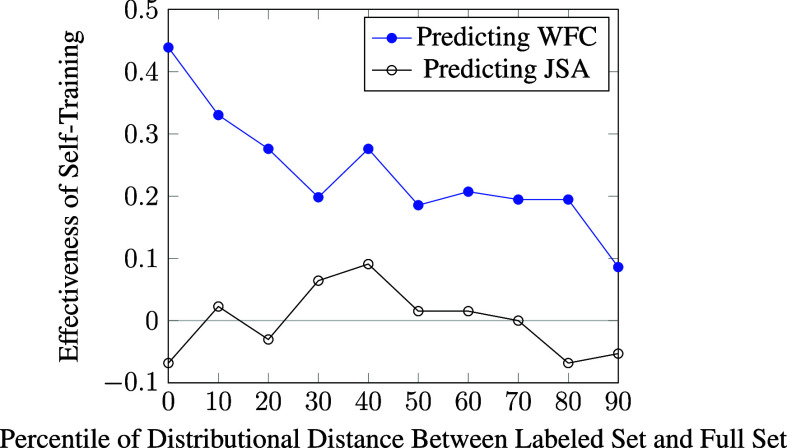
Relationship between effectiveness of self-training and representativeness of labeled set.*Note*: WFC, work-family conflict; JSA, job satisfaction. *Source*: Swiss Household Panel (SHP).

As can be seen from Figure 2, when self-training is used to predict WFC (i.e., to test the presence of WFC 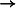 JAS), there is a clear, negative, correlation between its effectiveness and the distributional distance. In contrast, such correlation disappears in the prediction of JSA (i.e., testing of JAS 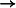 WFC), as the effectiveness of self-training hovers around zero regardless of the distributional distance. This clearly shows that, whereas the distribution of JSA reveals valuable signals for predicting WFC—meaning that WFC may have a causal effect on JAS—the distribution of WFC carries no information for predicting JAS, suggesting that the causal direction is unlikely to flow from JAS to WFC.

## 7 General discussion

In this section, we first discuss the research implications of our new method. We then review the limitations of our method and the future research needed to address them.

### 7.1 Research implications

For testing reverse causality, the main research implication of our semi-supervised learning based method is its ability to identify causal directions without presuming distributional or functional features of the data-generating process. Our method achieves this by leveraging recent advances in machine learning that directly link the causal direction with the effectiveness of semi-supervised learning. A unique feature of our method is that it is an umbrella algorithm independent of the underlying learning algorithm, which can be any algorithm for supervised learning. As such, researchers can freely choose a learning algorithm that fits the type and scale of their data before using our method for causal identification.

More broadly, the main research implication of our work is its transfer of insights of causal learning (Peters et al., 2017), in particular Schölkopf et al.’s (2012) seminal finding that links causal direction with the effectiveness of semi-supervised learning, into the methodological arsenal of causal inference in psychology. Whereas most existing work in causal learning—perhaps owing to the disciplinary focus of machine learning—leverages this link to explain (Schölkopf et al., 2012) or improve (Kügelgen et al., 2019) the performance of machine learning algorithms, our contribution is to demonstrate that the exact same link can be used to develop a concrete method for identifying reverse causality, addressing long-standing challenges in the analysis of longitudinal data in psychology and other disciplines.

### 7.2 Limitations and future directions

#### 7.2.1 Capability of semi-supervised learning

The statistical power of our method depends on the capability of the semi-supervised learning algorithm in approximating the functional relationship between *X* and *Y*. On the one hand, this allows our method to generalize beyond linearity to allow nonlinear *X*–*Y* relationships. On the other hand, it also brings about the limitation that, if the semi-supervised learning algorithm being used cannot effectively approximate a nonlinear relationship between *X* and *Y*, our method may generate Type II errors.

In the main simulation study in our current work, we employed a linear model (i.e., CLPM) as the underlying data-generating process. This choice allowed us to concentrate on the overarching design of our method rather than on fine-tuning the underlying learning algorithm, given that even a simple algorithm (like OLS) would likely suffice for a linear relationship. Nonetheless, although the theoretical foundations of our method extend readily to more complex, nonlinear relationships between *X* and *Y*, we did not evaluate the performance of our method in the presence of such relationships. As a natural next step, future research could provide a comprehensive empirical assessment of the statistical power of our method across a wider range of data-generating processes, encompassing both linear and nonlinear scenarios beyond those considered in this study. Additionally, exploring various underlying learning algorithms and other semi-supervised learning designs will be valuable for capturing the inherent complexity of nonlinear relationships between *X* and *Y*.

In the longer term, recent advancements in machine learning offer two key insights. First, there are semi-supervised learning algorithms that offer *universal approximation* (Goodfellow et al., 2016), meaning that they are theoretically capable of approximating any arbitrary function in a Euclidean space. Examples include the use of our method (i.e., self-training) with deep neural networks (Hornik et al., 1989) or variational Gaussian processes (Tran et al., 2016) as the underlying learning algorithm. Second, unfortunately, one would have to restrict the type of relationship between *X* and *Y* in order to translate such theoretical feasibility into any practical guarantee. To understand why, consider a stylized example where *Y* is the encrypted value (i.e., ciphertext) of *X* based on a secret key. Theoretically, it is feasible for a machine learning algorithm to eventually learn how to predict *Y* from *X*. In practice, doing so constitutes a brute-force ciphertext-only attack against the encryption algorithm, which is commonly believed to be practically infeasible (Goldreich, 2004).

#### 7.2.2 Leveraging other advances in machine learning

A central premise of our method is the link between causal direction and the predictive accuracy of machine learning algorithms. When the causal direction flows only from *X* to *Y* (i.e., 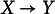 ), the distribution of *X* would reveal no information about the *X*–*Y* relationship, rendering semi-supervised learning ineffective.

Following the same logic, causal direction could also affect the effectiveness of machine learning algorithms besides semi-supervised learning. For example, a common generalizability issue facing supervised learning, covariate shift (Sugiyama et al., 2007), arises when a machine learning model degrades in predictive accuracy because of the distributional differences in predictor variables (i.e., *X*) between the training dataset and the dataset actually in need of prediction. As discussed before, it would be impossible for covariate shift to arise if the causal direction flows only from *X* to *Y* because, in this case, a change of *X*’s distribution should have no bearing on the *X*–*Y* relationship. In machine learning, Kügelgen et al. (2019) leveraged this property to address the degradation of predictive accuracy caused by covariate shift. Similarly, future research could examine the use of covariate shift or, more broadly, the generalizability of supervised learning models to help identify the causal relationship between *X* and *Y*.

#### 7.2.3 Integration of our method with existing methods

A key feature of our method is robustness to model misspecification, as it imposes no restrictions on the functional form of the data-generating process (e.g., linearity or the addition of independent noise). This flexibility minimizes the risk of spurious discoveries, such as the incorrect inference of reverse causality, caused by inappropriate model specifications or dependency structures. However, a notable limitation of our method lies in its relatively weak statistical power. For instance, our simulations demonstrate that it may struggle to detect reverse causality when the sample size is small. As such, our method may be better suited to larger datasets (e.g., with 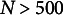 ).

These characteristics make our method a valuable complement to existing approaches, such as additive noise models and DDA, which can be sensitive to model misspecifications (e.g., Schultheiss & Bühlmann, 2024; Thoemmes, 2015). For example, while existing methods may not be ideally suited for exploratory investigations into causal directions between variables (Wiedermann & von Eye, 2015), our method can serve this purpose effectively. Researchers could first employ our approach as an exploratory tool to identify promising relationships. Subsequently, they may leverage existing methods to conduct confirmatory analyses of those relationships supported by robust theoretical formulations but not identifiable through our approach (e.g., in cases where neither 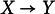 nor 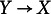 is detected).

Our method can also be seamlessly integrated with panel models such as CLPM and its variants. To illustrate, applying our method to infer the causal direction between *X* and *Y* could result in one of four possible outcomes: (1) the detection of 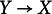 but not 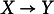 , (2) the detection of 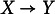 but not 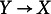 , (3) the detection of both 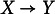 and 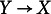 , suggesting reciprocal relationships or the potential existence of an unobserved confounder, and (4) the detection of neither 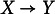 nor 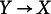 . With the first two outcomes, our model informs the model specification for CLPM, as researchers could choose to remove the cross-lagged effect inconsistent with the outcome of our method. An important future direction for research is to study whether causal learning could be used not only for identifying the causal direction (as in our work) but also for estimating the temporal lag of an effect, which would further improve the model specification for CLPM. The third outcome could prompt researchers to consider panel models that allow for certain types of latent confounders—e.g., the RI-CLPM (Hamaker et al., 2015), which models time-invariant confounders, or Latent Curve Models with Structured Residuals (LCM-CR; Curran et al., 2014), which models time-varying confounders with patterns such as autoregressive structures. Finally, as discussed earlier, researchers facing the fourth outcome could consider the use of existing confirmatory methods, e.g., additive noise models and DDA, to identify the causal direction.

## 8 Conclusion

In conclusion, our work proposes a novel method that integrates machine learning and reverse causality testing. Through mathematic analysis, simulation studies, and a case illustration, we demonstrates the effectiveness of this method. We hope this approach inspires future research to more strongly embrace the advancements in computer science and machine learning to enrich the methodological toolkit of psychological sciences.

## Footnotes

1 Throughout this article, we use “correlation” in the broader sense to refer to any (i.e., linear or nonlinear) co-variation (of two or more variables, constructs, etc.) that manifests in an observed dataset.

2 Such degrees of departure are typically quantified using higher-order central moments, such as skewness and kurtosis.

3 While outside the scope of this article, this exact inference also works when *Y* is not binary but follows other distributions such as Poisson (due to Linnik’s theorem; Linnik, 1957). Interested readers could refer to the rich literature of random variable decomposition (Lukacs, 1970) and the more recent literature of additive noise model (Hoyer et al., 2008; Peters et al., 2014) which are both intimately related to the unique identification of  (and thereby  and  ) given  .

4 As discussed earlier, see Daniusis et al. (2010) and Janzing & Schölkopf (2010, 2015) for formal definitions.

5 For simplicity in notation, this section uses *f* to denote the machine learning estimate, replacing the earlier notation  . This causes no ambiguity, as the ground-truth value of *f* is not discussed in this section.

6 Without loss of generality, we follow the convention in computer science to assume a base of 2 for all log operations, so as to measure entropy in binary bits.

7 We also tested alternative distributions (e.g., Gaussian mixtures) and observed no qualitative differences.

8 We also ran the SVM implementation and found qualitatively identical results.

9 We also implemented RI-CLPM in R with the lavaan package, replicating the setup from Mulder & Hamaker (2021). Results were consistent with those reported in Table 3.

10 Note that this means the use of unlabeled set actually *increased* predictive error in the majority (63%) of runs, likely due to the aforementioned sensitivity of SVM to outliers in the (uninformative) unlabeled set (Debruyne, 2009).
